# Towards User-Centred Prosthetics Research Beyond the Laboratory

**DOI:** 10.3389/fnins.2022.863833

**Published:** 2022-04-14

**Authors:** Hannah Jones, Lynda Webb, Matthew Dyson, Kianoush Nazarpour

**Affiliations:** ^1^Edinburgh Neuroprosthetics Laboratory, The University of Edinburgh, Edinburgh, United Kingdom; ^2^Intelligent Sensing Laboratory, Newcastle University, Newcastle upon Tyne, United Kingdom

**Keywords:** upper limb prosthetics, co-creation, policy, remote data, user-centred research

## Abstract

The purpose of this study was to explore a range of perspectives on how academic research and clinical assessment of upper-limb prosthetics could happen in environments outside of laboratories and clinics, such as within peoples’ homes. Two co-creation workshops were held, which included people who use upper limb prosthetic devices (hereafter called users), clinicians, academics, a policy stakeholder, and a representative from the upper-limb prosthetics industry (hereafter called professionals). The discussions during the workshops indicate that research and clinical assessment conducted remotely from a laboratory or clinic could inform future solutions that address user needs. Users were open to the idea of sharing sensor and contextual data from within their homes to external laboratories during research studies. However, this was dependent upon several considerations, such as choice and control over data collection. Regarding clinical assessment, users had reservations of how data may be used to inform future prosthetic prescriptions whilst, clinicians were concerned with resource implications and capacity to process user data. The paper presents findings of the discussions shared by participants during both workshops. The paper concludes with a conjecture that collecting sensor and contextual data from users within their home environment will contribute towards literature within the field, and potentially inform future care policies for upper limb prosthetics. The involvement of users during such studies will be critical and can be enabled via a co-creation approach. In the short term, this may be achieved through academic research studies, which may in the long term inform a framework for clinical in-home trials and clinical remote assessment.

## Introduction

Research on upper limb prosthetics typically occurs within the controlled laboratory and/or clinical environments, which do not reflect usage in the real world. The lack of real-world data from research studies may contribute to the limitations of current prosthetic devices in addressing user needs. Studies outside of a laboratory have focussed on the functional performance of an upper limb prosthetic device, but have gathered limited contextual data (Hargrove et al., 2017; Graczyk et al., 2018; Cuberovic et al., 2019; Brinton et al., 2020; Chadwell et al., 2020; Schofield et al., 2020; Osborn et al., 2021; Wu et al., 2022). The importance of gathering contextual data to inform decision- making has been highlighted within clinical practice and health policy sectors (Langlois et al., 2018). For instance, the National Health Service (NHS) England clinical commissioning policy for the provision of multi-grip upper limb prosthesis has identified the need for subjective and objective user data as evidence to inform future policies (NHS England, 2015). This evidence gap indicates a requirement to conduct research studies that gather real-world sensor and contextual data, with a view to creating advanced solutions that address user needs and impact policy within the field.

A user-centred approach to research on prosthetics can lead to solutions that address user needs effectively (Biddiss et al., 2007; Laffranchi et al., 2021). The term “user-centred” refers to users at the centre of a process. To ensure a user-centred approach, it is important to involve users throughout the research process. Currently, there is limited literature on user involvement in upper limb prosthesis research that occurs outside of the laboratory (Jones et al., 2021). Recent research has applied a range of methods that can facilitate a user-centred approach within a health-related context, such as workshops, focus groups, and questionnaires (Chandran et al., 2020; Ku and Lupton, 2020), and living laboratories (Favela et al., 2015). Studies have identified factors that researchers should consider, which may impact user involvement within research, such as power-sharing and mutual learning between all involved (Hickey, 2018; Tembo et al., 2021). Co-creation is an approach that can facilitate user involvement throughout the research process, such as contributing to study design, and disseminating findings (Jones et al., 2021). A co-creation framework involves co-ideation, co-design, co-implementation, and co-evaluation (Pearce et al., 2020).

This article presents the results of two co-creation workshops that aimed to: (i) gain academic, clinical, and user perspectives of the requirement for remote research and clinical assessment from the home; and (ii) gather user perspectives towards participating in remote research from the home. The term *remote* refers to people sharing real-time and/or recorded data from an external location (e.g., a home) to a researcher or clinician at a laboratory or clinic. With the term “contextual data” we refer to information on the psychological, social, and environmental context in which a person uses their prosthesis within their home, beyond the functional metrics that are recorded routinely as part of an experiment. The views from workshop participants are documented within the findings.

## Materials and Methods

The two co-creation workshops were approved by the local Research Ethics Committee at the School of Informatics, The University of Edinburgh (Ref: 2019/89177).

### Workshop Design

**FIGURE 1:**
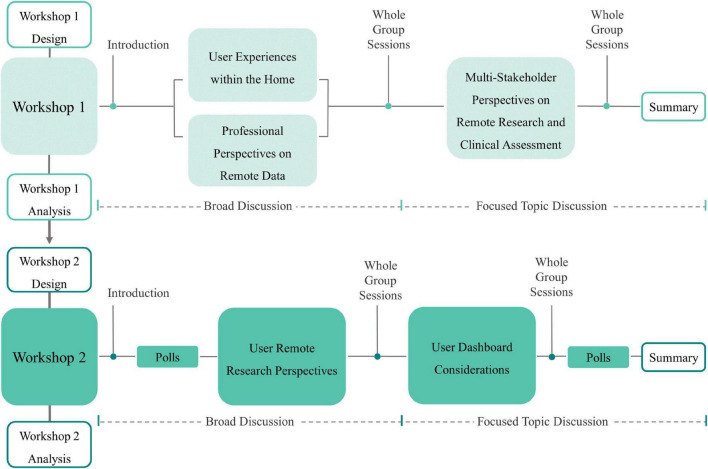
Overview of the structure of Workshops 1 and 2.

The workshop design included sections where the participants were all together and sections that took place in smaller break-out groups of five participants or less. The first half of the workshops facilitated discussions around a broad topic providing an opportunity for participants to share their experiences and opinions. The second half of each workshop focussed on a specific area within the original broad topic. Each break-out group shared a top-level overview to all workshop participants during the whole group sessions. Figure 1 illustrates the structure of the workshops.

The research team assigned participants to each break-out group considering factors such as stakeholder group, gender, and known prior academic and/or clinical interaction. Each group had a facilitator, an observer, and a note taker. Due to the COVID-19 pandemic, both workshops were held online using Microsoft Teams. Participant contributions were captured by notetakers on a digital whiteboard, called Mural (Supplementary Material A).

### Workshop 1

Workshop 1 explored the first aim of the study: gain academic, clinical, and user perspectives of the requirement for remote research and clinical assessment from the home.

Table 1 presents the participant groups for Workshop 1. Two groups were formed for the first half of the workshop: (1) Users, and (2) Professional (clinicians, academics, and industry).

**TABLE 1:** Workshop 1 groups.

		User perspectives	Professional perspectives		
		User	Clinician	Academic	Industry
**First Half**	Group 1	3	0	0	0
	Group 2	0	3	2	1
**Second Half**	Group 1	1	1	1	1
	Group 2	2	1	2	0

Each group discussed the following questions:

• Group 1: *What are the everyday challenges that you [users] experience in the home?*
• Group 2: *What information would be useful to know from users in their home?*

In the second half of the workshop, new groups were formed and a mix of both users and professional stakeholders were in each group. The following question was explored by both groups:

• *If a bridge can be made between the home environment and the clinician, what information should be exchanged and how?*

### Workshop 2

The design of Workshop 2 was informed by the analysis of the data gathered in workshop 1. Workshop 2 explored the second aim of the study: gather user perspectives towards participating in remote research from the home. Table 2 presents the participant groups for Workshop 2. Users, an academic, and a policy stakeholder were in attendance.

**TABLE 2:** Workshop 2 groups.

	User perspectives	Professional perspectives	
	User	Academic	Policy
Group 1	2	0	0
Group 2	4	0	1
Group 3	3	1	0
Group 4	2	0	0

First the user participants completed a poll of six questions about the type of information they would be willing to share in a research study (Supplementary Material B). Then participants broke into the small break out groups to discuss the following open-ended question:

• *In a research study, what would you want to know, what information would you be willing to share, what would be valuable to share and how?*

The second half of the workshop explored how data could be shared between stakeholders during a research study, through a user dashboard. Each group explored the following questions:

• *What would you like to see in a user dashboard?*
• *How would you like to interact with a user dashboard?*

After the discussions, the users repeated the same poll from the beginning of the workshop.

### Workshop Analysis

The workshop recordings and Mural boards were reviewed, and topics of discussion were identified. The open-ended questions were used as a basis to structure the reported findings, with topics of discussion presented as sub-headings within the section “Results.” The analysis was informed by a thematical approach, as described by Braun and Clarke (2006) and Staniszewska et al. (2018).

## Results

The results are presented in the order of which the questions were asked in each workshop. Direct quotations from the workshop participants are shown in *italics*.

### Workshop 1

#### User Group: What Are the Everyday Challenges That You Experience in the Home?

Users identified challenges that they experienced when carrying out everyday activities within the home. These challenges predominantly occurred in the kitchen and the bathroom. The challenges were around self-care activities, including getting dressed, bathroom-based activities, and taking medication. In addition, eating with cutlery was shared as a common challenge for all users.

Most users shared how they refer to their prosthetic devices as “*tools,”* rather than an extension of themselves. The users trust in using a prosthesis depended upon the task they were trying to carry out. For example, draining a pan of boiling water was an activity whereby trust of a prosthesis was limited, with one user sharing that they would rather use their stumps. The users shared how they lacked trust in their prosthesis, which often led to finding alternative ways of conducting tasks, e.g., “*I constantly have to find ways around*.” The discussion also explored the need to forward-think before conducting a task, which one described as “*exhausting.*”

Users were open to the idea of sharing information with clinicians regarding their everyday challenges. For example, one user would share the challenges when using touch screen devices and the importance of compatibility between a prosthesis and a device, e.g., a mobile phone or tablet. To that end, the user stated how the ability to communicate via such devices has an impact upon their daily lives: “*when I am at home on my own, my computer [or touch screen device] is my main way of interacting with people outside.”* Users would share the challenges faced when eating meals requiring cutlery, as they identified how this impacted upon their comfort to eat within their own home, and eating out at friends or family homes, or restaurants.

#### Professional Group: What Information Would Be Useful to Know From Users in Their Home?

Clinicians within the group shared that it would be useful to receive data on what grasps people use to carry out everyday activities within the home. Clinicians shared how receiving such data may improve efficiency when users try multiple devices, as the data could inform the decision on which prosthesis(es) to prescribe. This could be possible by building an awareness of why and how people use a prosthesis outside of the clinic, compared to the intended use that is outlined during assessments, which, as one clinician shared: may be influenced by eligibility criteria, such as NHS prescription.

The notion of conducting broader clinical assessments was discussed. An academic stated that they could see a benefit in conducting assessments that incorporate multiple sources of data, which could provide a more rounded view of how people interact and use a prosthesis. To that end, collecting user contextual data was discussed in addition to functional data. One clinician shared: “*[currently the assessment is:] ‘can someone pick up a glass of water and take a drink without spilling the water – yes/no?’ The assessment does not take other forms of information into account: was it comfortable, did you do the task as quick as you wanted to?”* The clinician shared how contextual and functional user data could inform prescription decisions, and more broadly it could enhance clinical services and devices. Furthermore, another academic shared how assessment within peoples’ homes is a version of a controlled environment and data should be gathered from beyond the home, where people interact within their communities.

Challenges were raised by a clinician regarding the practicalities of conducting clinical assessments outside of the clinic that include functional and contextual data collection. The clinician stated: *“It may be a better outcome if somebody is being assessed within the environment, they are going to use the equipment, [*…*] but that requires additional support and resources.”* Furthermore, they shared challenges of the scalability of assessment outside of a clinic. To that end, they scoped out the possibility of translating findings from small scale in-home trials to laboratory-based settings, so that clinical assessments could move towards incorporating more real-life scenarios for all users: “*nice to have the real home situation, so we can replicate situations in the lab.*”

Gathering longitudinal data was discussed within this group. One academic stated how there is currently limited longitudinal data within the field of research on upper limb prosthetics, which impacts upon the ability to foresee how peoples’ needs change over time. Furthermore, the academic stated when conducting individual goal-led strategies over an extended period, consideration should be applied to the flexibility in how goals are defined and how they evolve as the user ages.

#### Multi-Stakeholder Group: If a Bridge Was to Be Made Between the Home Environment and the Clinic: What Information Should Be Exchanged and How?

##### Current Communication Between Users and Clinicians

Information exchange between users and clinicians occurs during clinical appointments according to all participants. One user stated they would contact a clinician between appointments when they had a problem with their device, however, if such a scenario did not occur, then a clinician did not contact the user between appointments. Another user said how they would like to be able to share with their clinician their “*life experience*” data such as how they have adapted to use their prosthesis, but currently they “*haven’t been able to cascade that back to the clinic*.”

##### Defining the Study

The importance of identifying the purpose of data collection was discussed. Two clinicians and an academic highlighted the need to clarify what the information would be for, selecting the questions that need to be asked, and identifying the appropriate data collection method, e.g., one clinician shared: *“There needs to be a defined question that we need data to enable us to answer*. *If a stream of data would help us problem solve, then that would be useful.”* An academic also shared how a balance must be established between what information is useful from a research or clinical perspective, and what data is acceptable to collect from a user viewpoint.

##### Sharing Remote Data

Perspectives on sharing data with clinicians from a home environment were shared. All users stated how they would be willing to share data collected from their home with clinicians, e.g., “*happy to share anything about how I use them* [prostheses].” This user perspective was congruent with a clinician, who stated that it would be beneficial to have a system whereby the clinical team could conduct remote diagnostics of the prosthetic device. This could lead to mitigating device issues before they materialise.

Methods that enable users and clinicians to share and receive functional data were discussed, for example: sensors that remotely collect and transmit data on a prosthesis. A user shared: *“Sensors are fine. I don’t mind – whatever is going to produce the most useful data*.” They also shared an idea that changes to a prosthesis could be applied remotely, which they stated: *“that would be amazing.”* However, two users were reluctant to the idea of remote data streaming from a prosthesis sensor to the clinic. Concerns were raised about the potential of prosthesis usage data to influence device prescription.

When discussing users’ perspective on sharing functional and contextual data with an academic team as part of a research study, one user shared their willingness to be involved: “*I am helping to develop something for the future. So, it doesn’t have to change for me, because I am influencing what the final product would be.*”

Perspectives on sharing contextual data were shared. An academic believed that contextual assessments could incorporate the environmental context of user scenarios: *“I think it is a fantastic idea to have a more environmentally contextualised assessment, because device usage does not happen in a vacuum – it happens in a context. The elements within that context can make it easier or very much more difficult, depending on the context of use.”* Another academic raised privacy issues during the discussion, highlighting the importance of considering ethical considerations when collecting contextual data, for example, video recording within a home environment. Both academics highlighted potential implications that could arise if the data is shared with certain stakeholders, such as insurers, which could influence device prescriptions.

Methods that enable users to share contextual data were discussed. One user shared: “*I wouldn’t want a video following me around all the time. But for 1 day, I could do that [*…*] so have 1 day where I recorded everything that I did, and what tools [prostheses] I used. And how successful they were.”* In addition, an academic stated how consideration needs to be applied when assessing behavioural changes when a user is aware of data collection that is in progress.

##### Receiving Remote Data

The practicalities of receiving user data from a clinical perspective were discussed. One clinician highlighted the importance of identifying appropriate metrics to use when interpreting the data. For example, they shared that “*time usage is not always the best measure.”* This point was referring to a scenario whereby a prosthesis may be used 2 days per week, which from a user perspective, could be critical in assisting the individual and the broader impact upon quality of life. Resource and funding constraints were discussed by the clinicians in relation to providing a service that could remotely collect and receive data, e.g., a clinician shared: “*Having a continuous stream of data: I am not sure what I would do with that from a clinical point of view; when I am trying to balance lots of things.”* Furthermore, another clinician stated: “*the data needs to be in a useable format for the clinician to use*.”

##### Two-Way Communication

The notion of a communication loop between clinicians and users was discussed by all stakeholders. From a user perspective, sharing data would only be useful if they knew how it would contribute to service improvements or research studies. For example: a user could share a concern by sharing data before a clinic appointment, which could give the clinician time to address a situation remotely, and/or be more informed going into an in-person appointment. The industry stakeholder shared how circular communication with users can lead to iterative development of the overall design and functionality of a prosthetic device.

### Workshop 2

#### In a Research Study What Would You Want to Know, What Information Would You Be Willing to Share, What Would Be Valuable to Share, and How?

##### User-Researcher Communication

The type of information users would like to receive from researchers during a study was discussed. The information users asked for included: before an experiment – clarity regarding the study duration, aims, and objectives; during the study – an awareness of other participants’ study experience; and after the study – a final report.

Methods that enable regular communication from the research team to the users were discussed. One user shared how they would like the opportunity to have video calls with the research team to discuss their experience of the study and share/receive updates through a conversation, as *“you start to wonder what is happening.”* The user also shared: *“I like [the idea] of feeding back information - it will keep people engaged – such as a newsletter.”* A user shared the idea of a secure online portal with a chat function, to enable experiences to be shared within the study community. Other users indicated that they would like to see a breakdown of their data: *“It is useful to know what I have been using [grips] throughout a period of a day.”*

Two-way communication was a common thread throughout all discussion groups, with a user highlighting a key question that should be answered through communication with the research team: *“How has the data I have provided helped you [researchers] to develop the tool?”* This user also shared that the relationship with the research team is important for engagement throughout the study: *“the relationship is one of the things that keeps you involved*…*so I am more engaged with the process of research, and I am more likely to carry on because of that.”* A policy stakeholder expressed the value of having two-way communication channels, as it can make the study more engaging for all involved. However, they recommended that care should be taken to ensure that communication exchange with users’ during the study does not affect the reliability and reproducibility of the results.

##### Types of Data Users Are Willing to Share

A range of functional and contextual data that users were willing to share within a research study was discussed.

A user expressed how they would share data about how arm positioning impacts the functionality of their device, and information about the pain that is experienced from pressure or rubbing of their prosthesis. They also stated that they would be willing to share functional data from a prosthesis, particularly when the device does not perform the intended grasp. Another user shared how sensors that detect compensatory movement could inform fitting appointments within clinics. Furthermore, a user expressed the value in viewing functional data during a study to inform an understanding of their own prosthesis usage: *“I still personally, because of my situation and experience, have a particular focus on – does it grip properly?”*

One group shared a view that current data sourced from users do not fully represent peoples’ lived experience. Users indicated that information about device satisfaction, daily activities, and quality of life could be useful for researchers. For example, one user shared that *“quality of life at the end of the day is the goal,”* and therefore would be willing to share that type of data. Another user shared how many factors play a role when choosing to use a prosthesis: *“It is such a complex, multi-faceted thing. Your identity, how you are seen in the world, and how you feel your limb represents you.”*

A number of users could see the value of sharing location-based data sourced from beyond the home environment. Users could foresee the benefit of such data to provide context to the information sourced from sensors. For example, a user shared that they would be willing to send functional data outside of the home, such as whilst doing activities at the gym.

##### User Data Sharing Methods

Methods that enable users to share their data from their home with a research team was discussed. There was a notion that data collection methods need to be flexible to fit within peoples’ daily routines, as one user shared: *“A system that is very simple, but capable of elaboration.”* Elaboration was described as providing more detail about a specific task at a convenient time for the user, as people may not be able to record information about an issue when it is being experienced. To that end, a traffic light system was discussed, whereby users could log a time and location of activity, then provide more information at a later stage in the day: *“A flag-up would be a good idea.”* One group discussed how a traffic light system in combination with an online portal could cater for all participants; enabling people to provide more information on the online portal if they choose to.

A couple of users shared their views on writing, “*I don’t like it when I have to write a lot.”* One user related an experience of sharing written information during a research study: “*It was difficult to type out and convey the experience with words [of using a prosthetic device].”* The ease of recording and sharing data was a consideration that was discussed, such as uploading data directly to an app: *“rather than record it, save it, then send it.”* Two users shared how they were willing to share functional data from a device but were less inclined to provide audio and video recordings during a study.

##### User Data Sharing Frequency

The frequency of sharing or uploading data, manually or automatically was discussed. One user expressed their perspective on manually uploading their data: *“I am not sure I want to be obliged to sit down every night and upload data to you [researchers].”* Whilst another user stated: *“For me I have already a lot of things to do, so maybe if you could do it automatically, it would be ok*.” Another user shared how they would be willing to participate in automatic uploads, in addition to responding to questionnaires.

A user preferred to have an opportunity to discuss and share qualitative data with the research team *“on a monthly, or two monthly bases.”* Time commitment was discussed by users within two groups. A user shared that: *“time is finite,”* and that the frequency of collecting user data needs to consider *“the most economical use of time”* for the participants.

##### Users’ Understanding of Ethics

Ethics was raised by users who understood the concept of informed consent. They stated the importance for research participants to understand the purpose of a study, how their contributions will inform the research, and how the information will be disseminated during and after a study: *“It is important to get the right ethical options made clear.”*

Users highlighted the need for clarity on what type of data will be collected, especially from within a home environment when providing context. For example, if recording a video, a person may capture a wider context than intended, such as items in their home, and family members. Users were also aware of what constitutes as data within a research study: *“I think any information we provide to the research team is data.”*

The topic of reward within research studies was discussed. One user shared: “*will there be people who want to be financially incentivised? [*…*] per hour fee of data?”* In addition, one group briefly discussed intellectual property ownership and involvement of users within the research and development process. One user shared the following in that regard: *“[what if] There is an early-backer discount if we want to be involved in the technology in the future.”*

#### User Perceived Information Requirements for a Remote Research Study From the Home

This part of the workshop explored how people would want to experience a remote research study from the home, and ways they could communicate with a research team via a digital interface. The following two questions were asked and discussed sequentially during this part of the workshop:

What would you like to see in a user dashboard?
How would you like to interact with a user dashboard?

##### Data Representation

In the context of a dashboard: users shared that they would like to choose how their sensor and contextual data would be represented.

Two users stated how they would prefer graphical visualisations such as a pie or bar chart, rather than detailed analysis of sensor data. An idea of a timeline was also discussed, whereby users could upload contextual data in relation to a specific activity during the day: *“I really like the idea of a very visual diary, in addition to the data about the grips*…*something that tells you where you were, or what you were doing.”* One user shared: *“I like the idea of a timeline that could be annotated in various ways maybe you could attach a video to a particular point to show what you are trying to do.”* One user stated they would like to see a visualisation of their muscle activity: *“I’d like to have something that recognises your muscle activity – whether I am overusing it, or under using it.”*

In the context of the end of a research study, written reports were also discussed with a consensus that summaries would be optimal, with the option to read detailed reports: *“It is better to have an executive summary, rather than the full report, sometimes.”*

##### Platform and Interaction Options

Sharing both sensor and contextual data via a platform, such as a dashboard was discussed, with one user sharing: *“finding a way to do that seamlessly would be awesome, because as we have said – that is what is missing.”* A user and the policy stakeholder highlighted the importance of being able to share videos to provide contextual data, as sensor data may not indicate the intention of the grasp. The user of that discussion gave an example of this, relating to how the quantity of grip types they used, did not represent the functional value of their prosthesis to them: “…*there is a functional level of body language communication that I was applying to use the hand. And that is one of the main things I do with it. If you counted the quantity of grips that were functional, it is probably less than 10%.”*

Physical interaction with an electronic device, such as a mobile phone or tablet was discussed in each group. Touch screens were a challenge for a number of users. One user described their experience of using their phone and iPad as *“difficult.”* Another expressed: *“I wouldn’t particularly like to have an app on my mobile phone [*…*] a computer for me is much easier.”* One user, used a stylus to interact with their tablet, they proposed that a tick-box question and answer function could work – *“so that you don’t have to write.”* There were differing opinions on voice interaction, one user shared that *“voice recognition is wonderful when you are on your own, with no other sounds,”* three other users shared how they would use an audio recording function, with one user stating: *“I need to have voice control.”*

##### Workshop 2 Polls

**FIGURE 2:**
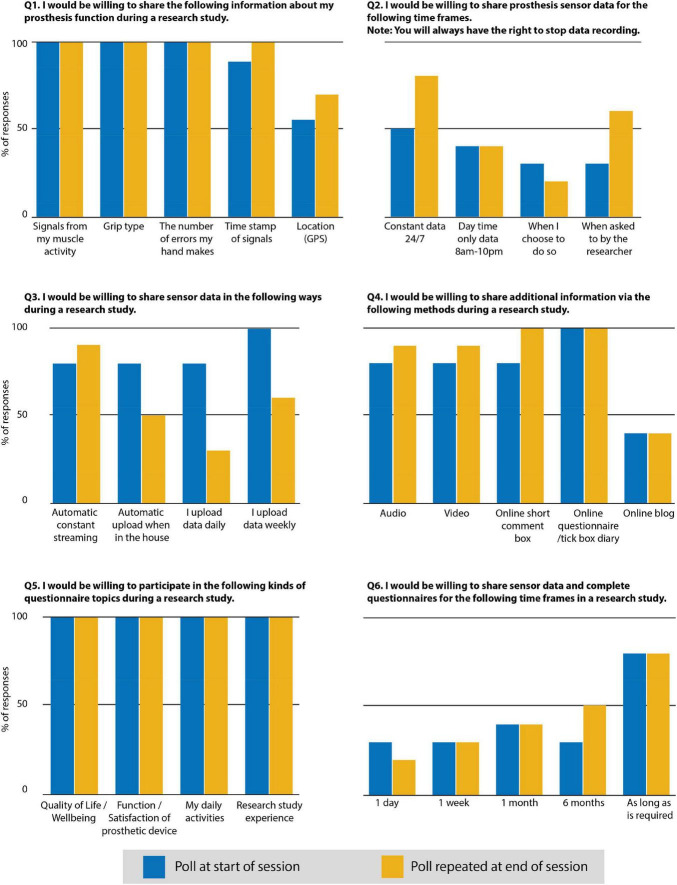
Results of the polls completed at the beginning and the end of Workshop 2.

The workshop polls were completed by user participants at the beginning and the end of the workshop. The multiple-choice questions allowed for multiple answers. The results of the polls are presented in Figure 2.

Responses to the polls showed an overall willingness to share data and engage with research. The change of responses between the first and second poll was in the direction towards a greater willingness to share data during a research study. In particular: willingness to share location (GPS) data increased from 55 to 70%; sharing data 24/7 increased by 30%. The following all increased by 10%: time stamping of data signals, automatic streaming of data, video, and audio data. Willingness to participate in a study for a 6 months duration increased from 30 to 50%.

## Discussion

This paper presents findings from two co-creation workshops that explored how academic research on upper limb prosthetics and clinical assessment could happen in less controlled environments, such as within peoples’ homes. Workshop 1 aimed to gain academic, clinical, and user perspectives, as such the participants comprised a combination of users and professional stakeholders, including clinicians, an industry specialist, and academics within the research field of upper limb prosthetics. The aim of workshop 2 was to gather user perspectives towards participating in research from the home, remote from both the laboratory and the researchers.

Upon an agreement between stakeholders on the research and clinical value of data collection, users within both workshops shared an interest in participating in remote academic research, providing considerations are addressed, such as choice and control over what and how data is shared throughout a study. As such, when implemented, this paradigm could inform the future direction of academic research and clinical delivery by understanding how people use their prosthesis via the collection of remote sensor and contextual user data.

The discussions during workshop 2 explored user requirements for sharing remote sensor and contextual data via a user dashboard. Recent studies have used standard methods such as take-home dairies and quality of life surveys to collect additional data about prosthesis use (Graczyk et al., 2018 and Cuberovic et al., 2019). Findings from the workshops in this study indicate that users are willing to share more in-depth contextual data about prosthesis use and its effect on their wellbeing. Methods such as location GPS, video/audio recording, and online questionnaires were identified. The authors advocate that an Internet of Things (IoT) approach could be used to consolidate these multi-modal data gathering and facilitate a user dashboard (Wu et al., 2022). In this regard, privacy is a key consideration for user involvement, where public and private border lines could be complex (Atlam and Wills, 2020). Research into ethical principles for designing health-related IoT studies highlights that user privacy should be the first consideration and that user control on how data are shared is critical (Mittelstadt, 2017). Ethical documentation, such as participant information sheets, can assist in clarifying choice and control and may manage user expectations regarding involvement within a study. Furthermore, conducting ongoing consent, which could be obtained at multiple stages of a study, may contribute towards long-term user involvement (Grant et al., 2019).

Building relationships between users, researchers, and clinicians was a key discussion topic during both workshops. In particular, the need to establish relationships through regular interactions and forming trust between all involved. If these points are addressed, this could enable user involvement throughout longitudinal remote research studies. Co-creation can provide a platform from which such involvement can be realised. Several factors contribute towards co-creation, such as power dynamics between users and researchers. This is especially pertinent when considering how to enable shared decision making and mutual learning between all involved (Tembo et al., 2021). Implementing such principles within research studies can be a time-intensive process (Tembo et al., 2021) and may conflict with short-term academic research funding cycles. However, challenges may arise if such principles are not followed, especially when conducting collaborative research by building relationships between researchers and users (Greenhalgh et al., 2016).

The involvement of users within the workshops was based on a co-creation approach that enabled discussion and collaboration between all involved. A topic of discussion that organically emerged during both workshops was the importance of clarifying what involvement within a study entails from a user perspective, and how that can influence willingness to participate. The poll results during workshop 2 suggest that involvement in the co-creation workshop discussions may have informed peoples’ understanding of the context of a remote research study and what the data could be used for. This can be associated with the response to the second poll, regarding changes to what people were willing to share, the frequency of which data could be transferred, and the overall extended length of time people were willing to participate within a research study.

Discussions within Workshop 1 touched upon resistance from users on sharing remote data with clinicians, due to concerns that such data may influence prescription decisions, especially in countries such as the United Kingdom that have a national health system. However, clinical and user perspectives during the workshop identified that if contextual data is also recorded, then a broader assessment of how a device impacts upon quality of life may be established, leading to a more informed health and care service. From a clinical perspective, concerns were raised during Workshop 1 regarding resource capacity and technical feasibility to conduct remote clinical assessments. However, digitised health services have been implemented and will continue to expand over time due to the COVID-19 pandemic (Visram et al., 2020). A key factor of this step-change in health services is the access to appropriate digital skills training within the clinical sector (Maguire, 2020). This will be a critical aspect in a transition towards combining in-person and remote clinical assessment within the field. In addition, a digital interface and presentation of data in an accessible form is essential to facilitate and not hinder clinical service. Co-creation would be an important methodology to ensure the needs of the clinicians are addressed (Tang et al., 2018).

### Future Study Considerations

The study presents findings from a small sample size of stakeholders within the United Kingdom and Northern Europe (Workshop 1: 3 users, 6 professionals; Workshop 2: 11 users, 2 professionals). Recruitment for both workshops occurred via professional networks and social media. Interest in attending the workshops was high. However, as with all research engaging with the general public, issues can arise that lead to people needing to cancel their involvement due to health-related issues. These factors resulted in the number of attending stakeholders being less than the number of people who had signed up and consented to participate. Future studies should seek to involve a larger cohort of users to identify patterns and commonalities for conducting research in a home environment within a broader population size. One approach could be to run a series of workshops over a set period of time, which could accommodate larger participant numbers, whilst maintaining the capacity to deliver the workshops from a researcher’s perspective.

## Conclusion

This article reports findings from two co-creation workshops, which aimed to: (i) gain academic, clinical, and user perspectives of the requirement for remote research and clinical assessment from the home and (ii) gather user perspectives towards participating in remote research from the home.

The findings indicate that to better understand and serve the user population with prosthetic solutions that address user needs; both academic and clinical practice requires expansion beyond the laboratory and clinical environments into the daily lives of users. From the user perspective, it is important to consider their needs to maintain long-term involvement throughout a remote research study. To that end, users’ control over data collection, data privacy, and relationship between users and researchers are pertinent points that were raised within this study.

In the long term, academic studies that gather remote sensor and contextual user data could inform the delivery of clinical in-home trials, and care policies that outline the provision of clinical remote assessment. However, factors may impact the move towards this vision, such as the current short-term nature of academic research funding, and clinical resource and technical feasibility constraints.

The findings presented within the paper are indicators of current perspectives and future direction that is open to iteration, rather than absolute conclusions. Therefore, further co-creation studies are required to validate the findings and gather further data.

## Data Availability Statement

The original contributions presented in the study are included in the article/Supplementary Material; further inquiries can be directed to the corresponding author/s.

## Ethics Statement

The studies involving human participants were reviewed and approved by the School of Informatics Research Ethics Committee at The University of Edinburgh (Reference Number: 2019/89177). The participants provided their written informed consent to participate in this study.

## Author Contributions

HJ, LW, and KN conceived the study and oversaw the overall direction and planning of the workshops. HJ, LW, KN, and MD conducted the workshops. HJ and LW analysed the data and wrote the revised manuscript. HJ wrote the first draft of the manuscript. All authors discussed the results and commented on the manuscript revision.

## Conflict of Interest

The authors declare that the research was conducted in the absence of any commercial or financial relationships that could be construed as a potential conflict of interest.

## Publisher’s Note

All claims expressed in this article are solely those of the authors and do not necessarily represent those of their affiliated organizations, or those of the publisher, the editors and the reviewers. Any product that may be evaluated in this article, or claim that may be made by its manufacturer, is not guaranteed or endorsed by the publisher.
