# Supplementary material for: Towards User-Centred Prosthetics Research Beyond the Laboratory
Source: Front Neurosci. 2022 Apr 14;16:863833. doi: 10.3389/fnins.2022.863833 (PMC9048479; doi:10.3389/fnins.2022.863833)
Supplement: Supplementary file 1 [file Table_1.DOCX]

**Supplementary Material A: Mural Whiteboard: Workshop 1**

**
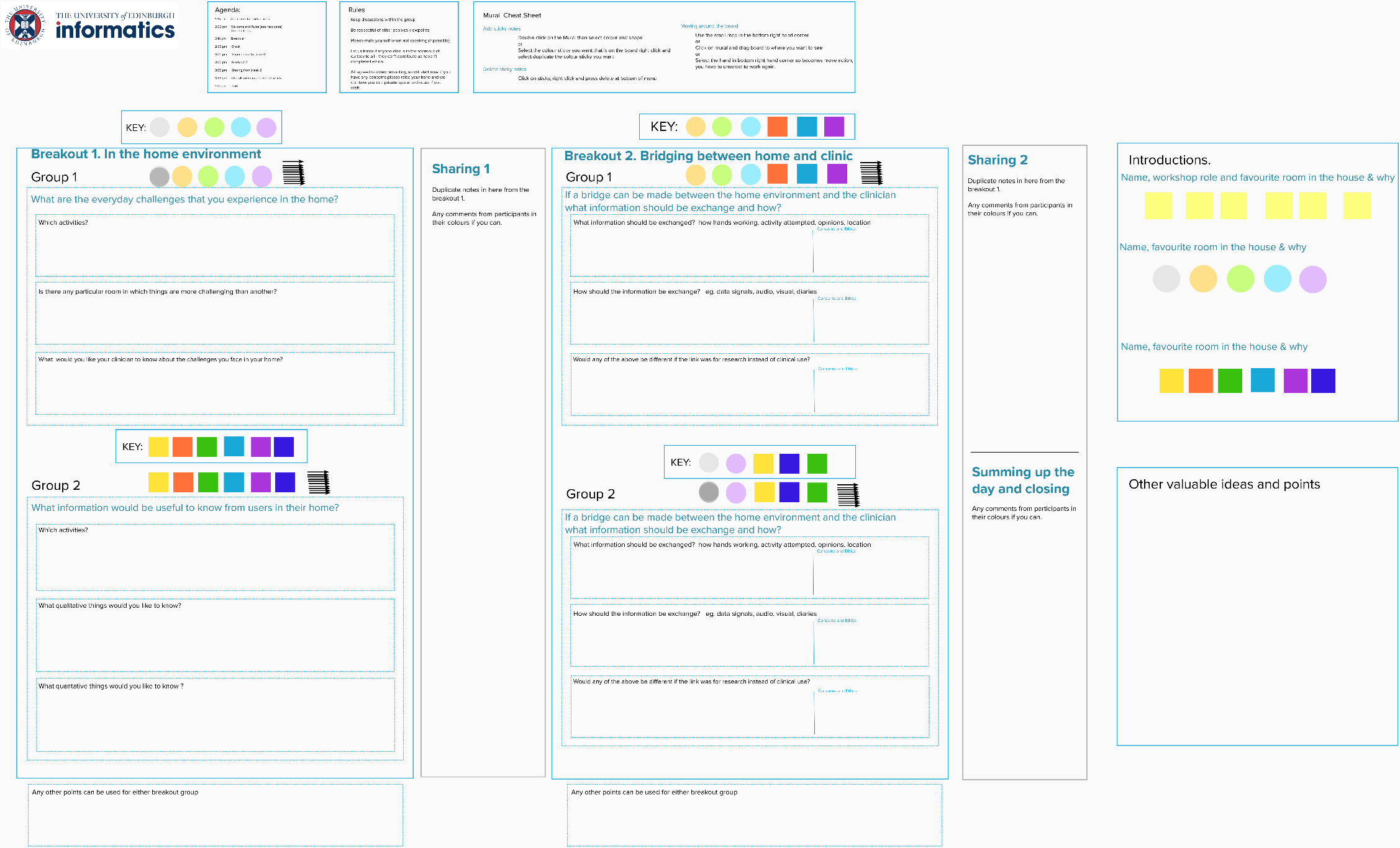
**

**Supplementary Material A: Mural Whiteboard: Workshop 2 First Half**

**
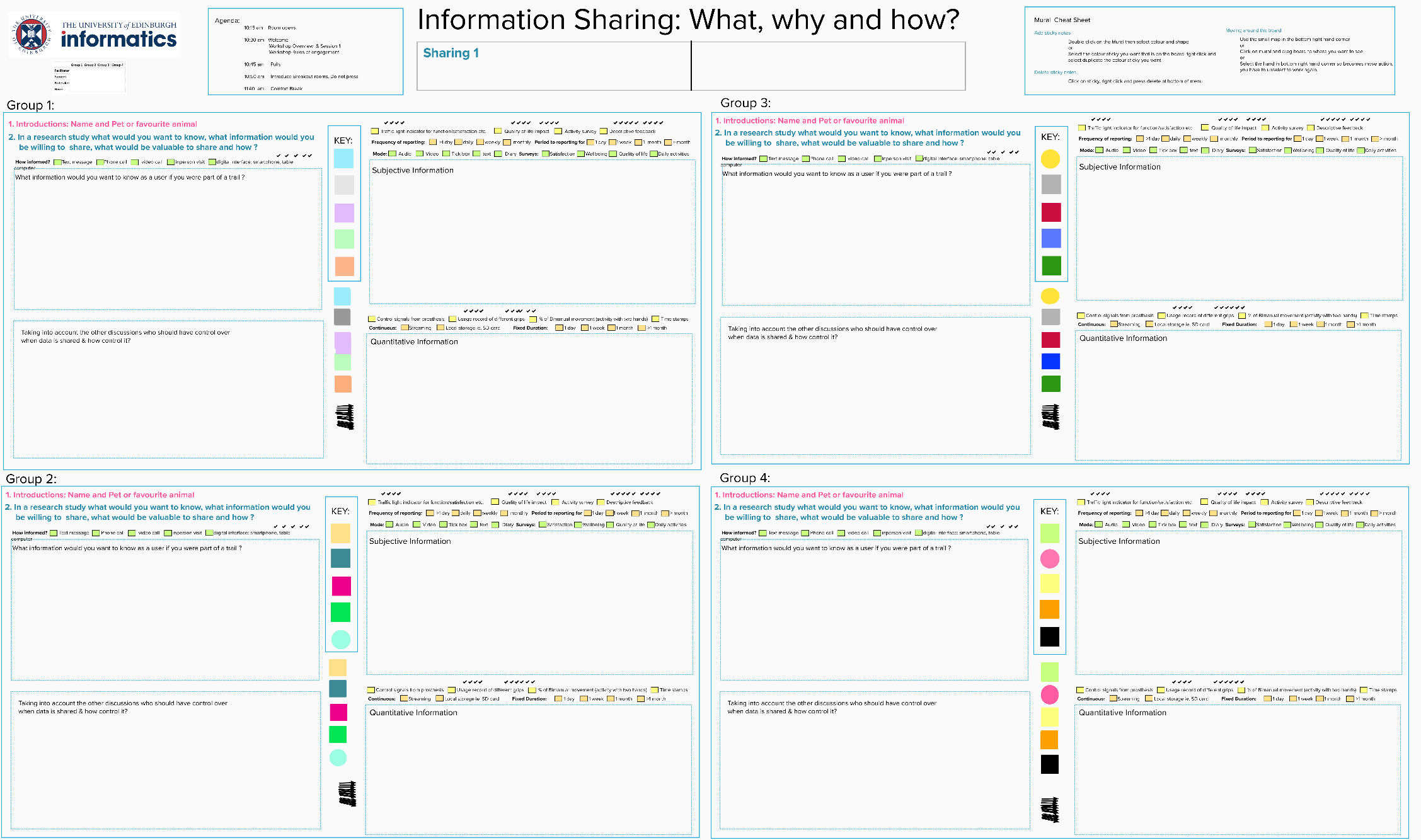
**

**Supplementary Material A: Mural Whiteboard: Workshop 2 Second Half**

**
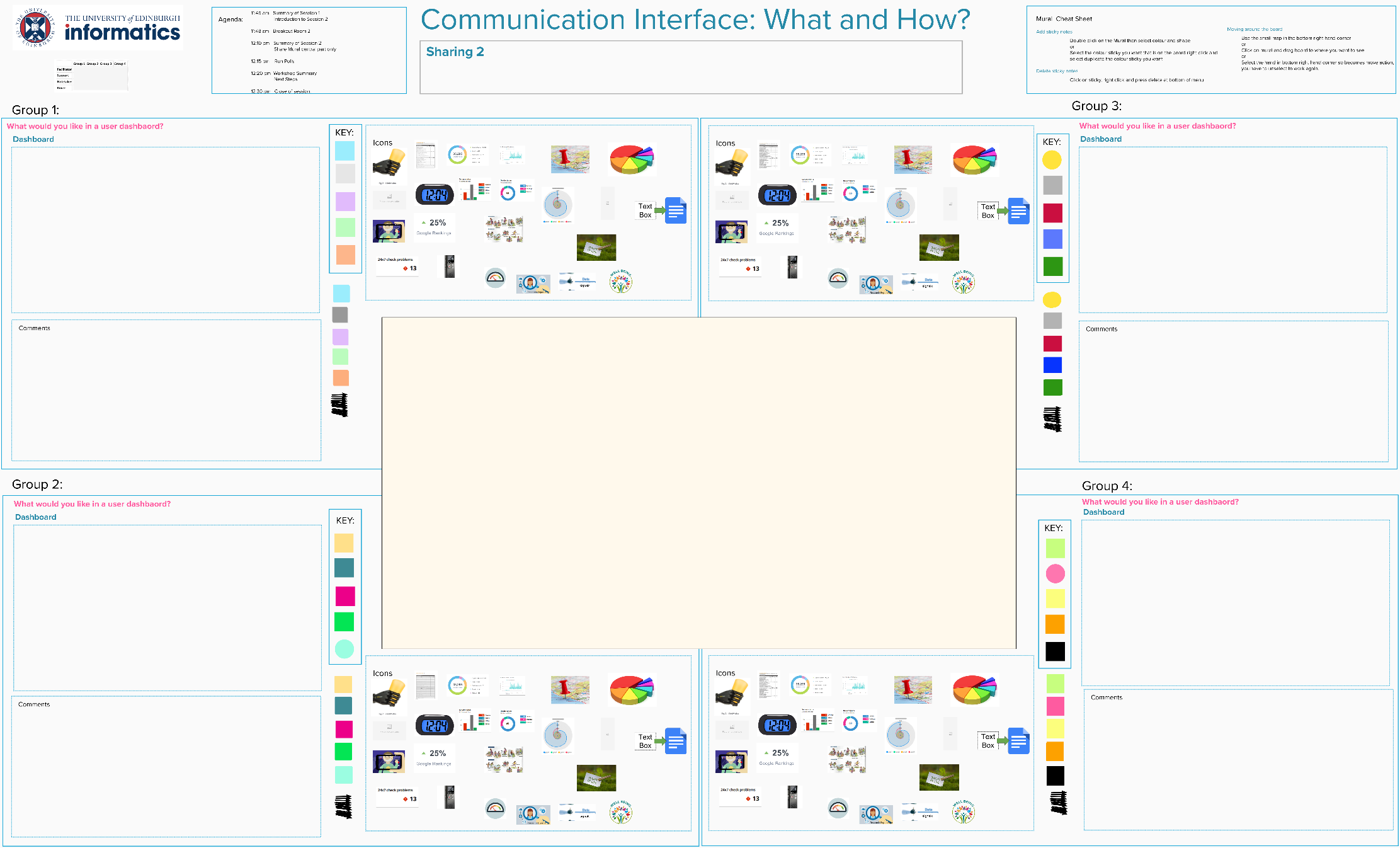
**
