# Supplementary material for: Towards User-Centred Prosthetics Research Beyond the Laboratory
Source: Front Neurosci. 2022 Apr 14;16:863833. doi: 10.3389/fnins.2022.863833 (PMC9048479; doi:10.3389/fnins.2022.863833)
Supplement: Supplementary file 2 [file Table_2.DOCX]

| Question | Multiple Choice Answers | | | | | |
| --- | --- | --- | --- | --- | --- | --- |
| I would be willing to share the following information about my prosthesis function during a research study. | Signals from my muscle activity | Grip type | The number of errors my hand makes | Time stamp of signals | Location (GPS) | None of the above |
| I would be willing to share prosthesis sensor data for the following time frames. | Constant data 24/7 | Day time only data 8am to 10pm | When I choose to do so | When asked to by the researcher |  |  |
| I would you be willing to share sensor data in the following ways during a research study. | Automatic constant streaming | Automatic upload when in the house | I upload data daily | I upload data weekly |  |  |
| I would be willing to share additional information via the following methods during a research study. | Audio | Video | Online short comment box | Online questionnaire / tick box diary | Online blog |  |
| I would be willing to participate in the following kinds of questionnaire topics during a research study. | Quality of Life / Wellbeing | Function / Satisfaction of prosthetic device | My daily activities | Research study experience |  |  |
| I would be willing to share sensor data and complete questionnaires for the following time frames in a research study. | 1 day | 1 week | 1 month | 6 months | As long as is required |  |
